# Cold tolerance in *Osmotin* transgenic tomato (*Solanum lycopersicum* L.) is associated with modulation in transcript abundance of stress responsive genes

**DOI:** 10.1186/2193-1801-2-117

**Published:** 2013-03-19

**Authors:** Vikas Yadav Patade, Deepti Khatri, Maya Kumari, Atul Grover, Sanjay Mohan Gupta, Zakwan Ahmed

**Affiliations:** Molecular Biology and Genetic Engineering Division, Defence Institute of Bio-Energy Research, Haldwani, 263 139 Uttarakhand INDIA

**Keywords:** *Osmotin*, Transgenic tomato, qRT PCR, Transcript expression, Free Proline, Ascorbate, Cold stress

## Abstract

Our containment trials have established cold tolerance in *Nicotiana tabacum osmotin* (*Nt Osm*) transgenic tomato (*Solanum lycopersicum* L. cv. Pusa Ruby). Though, the stress tolerance mechanisms have been studied at physio-biochemical levels, molecular mechanisms underlying the tolerant response are still not well studied. Therefore, quantitative transcript expression of *Osmotin* and other stress responsive genes (*CBF1*, *P5CS* and *APX*) was studied in response to cold (4°C; 2 and 24 h) treatment in the transgenic and wild type tomato plants. The expression analysis revealed differential transcript regulation in the transgenic and wild type plants on the cold exposure. In general, the genes were either earlier induced or the extent of fold change in transcript expression over the respective untreated controls was higher in transgenic than in the wild type plants on cold exposure. The transcript expression data also supported the metabolite analysis on free Proline and ascorbate content. The results thus suggest that constitutive over expression of the *Osmotin* modulate transcript abundance and functional expression products of the other stress responsive genes thereby, imparting cold tolerance in the transgenic tomato plants.

**Electronic supplementary material:**

The online version of this article (doi:10.1186/2193-1801-2-117) contains supplementary material, which is available to authorized users.

## Background

Osmotin or Osmotin-like proteins have been shown to be accumulated in response to various biotic and abiotic stresses in plants. Further, over-expression of the gene in transgenic plants is known to impart tolerance against various biotic and abiotic stresses including cold, salt and drought (Goyary 2009; Parkhi et al. 2009; Goel et al. 2010; Das et al. 2011; Subramanyan et al. 2011). Although there are some reports on the possible mechanism underlying the defense function of Osmotin against biotic stresses, its role in abiotic stress response is still unclear. Our laboratory has developed *Osmotin*-transgenic tomato using *Agrobacterium* mediated genetic transformation and advanced desired line to T8 generation to enhance cold tolerance. Further, containment evaluation trials at Defence Institute of High Altitude Research (DIHAR), Leh (11,500’ asl), India and Defence Institute of Bio-Energy Research Field Station, Auli (9,000’ asl), India have established cold tolerance in the transgenic lines (Goyary 2009). Mortality rate was considerably lower in transgenic lines than the wild types when the temperature dropped to −2.0°C. Our laboratory studies indicated that the cold tolerance in the transgenic is associated with significant increase in free Proline content and antioxidant enzyme activity in transgenic than the wild type plants on cold (4°C; 24 h) exposure (Goyary 2009). Similarly, other studies have also reported higher Proline accumulation *Osmotin* over- expressing transgenic plants on exposure to cold, salt, or drought stress (Parkhi et al. 2009; Goel et al. 2010; Barthakur et al. 2001), suggesting cross-talk between different stress response pathways. However, information on regulation of genes involved in various stress response pathways is not available in *Osmotin* transgenic tomato and other plants.

Recognizing the fact that stress response is built through a variety of altered mechanisms through transcript regulation of early responsive genes, regulatory genes, and effector genes (Mantri et al. 2010), we have undertaken a study to understand the molecular mechanisms based on transcript expression of stress responsive genes involved in various functions such as transcript regulation (*C repeat binding factors*: *CBF1*), osmotic adjustment (*Δ1- Pyrroline 5- Carboxylate Synthase*-*P5CS*) and antioxidant defence (*Ascorbate peroxidase-APX*) along with *Osmotin* in transgenic and wild type tomato plants in response to short-term (2 h) and relatively long-term (24 h) cold exposure. The transcript expression data is further supported with metabolite analyses on free Proline and ascorbate content in wild type and transgenic tomato plants on the cold exposure.

## Results

### Transcript expression analysis

The transcript expression of the genes was normalized using internal control (*18S rRNA*). Fold transcript expression of the genes in response to the cold exposure was calculated over the respective unstressed transgenic and wild type controls.

### Transcript expression analysis of *Osmotin*

**Figure 1:**
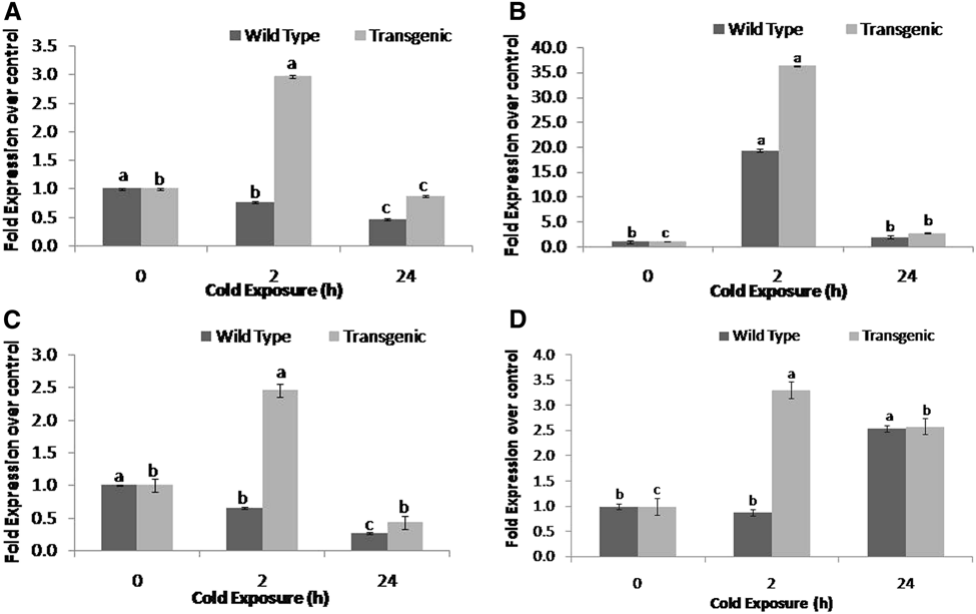
**Transcript expression analysis of*Osmotin*(Figure**1**A),*CBF1*(Figure**1**B),*P5CS*(Figure**1**C) and*APX*(Figure**1**D) in transgenic and wild type tomato plants exposed to cold treatment.** Plants at 90 days after transplanting were exposed to cold treatment for 2 and 24h and transcript expression was studied in leaves. The values are means (n = 4) fold expression over respective untreated control. Error bars indicate SE. The mean values in one series indicated by different letters are statistically significant (P ≤ 0.05) according to LSD test.

Transcript expression of *Osmotin* in transgenic plants increased significantly (P ≤ 0.05) in response to 2 h of cold exposure as compared to the untreated control. However, the transcript level again decreased in 24 h cold treated transgenic plants similar to that of untreated seedlings (Figure 1A). On the contrary, in the wild type plants, the fold transcript abundance over the untreated control was decreased in response to the cold exposure of 2 and 24 h.

### Transcript expression analysis of *CBF1*

Transcript expression increased significantly (P ≤ 0.05) in response to the short (2 h) cold exposure in transgenic as well as wild type plants (Figure 1B). However, the extent of increase was higher (36 fold) in transgenic plants than the wild type (19 fold). The fold transcript abundance again decreased in 24 h of cold exposure in both the transgenic as well as wild type plants.

### Transcript expression analysis of *P5CS*

The transcript expression of the gene increased significantly (P ≤ 0.05) on 2 h cold exposure in transgenic plants (Figure 1C). However, in response to the 24 h of cold exposure, the transcript expression decreased significantly than the untreated control. In wild type plants exposed to 2 or 24 h cold stress, the transcript expression was significantly decreased as compared to the untreated control.

### Transcript expression analysis of *APX*

The transcript expression increased significantly (P ≤ 0.05) in 2 and 24 h of cold exposure in transgenic plants as compared to the untreated control (Figure 1D). However, in case of wild types, the transcript abundance of the gene remained unchanged on 2 h cold exposure but increased significantly in 24 h of cold stress as compared to the untreated plants.

### Free Proline and ascorbate content

**Figure 2:**
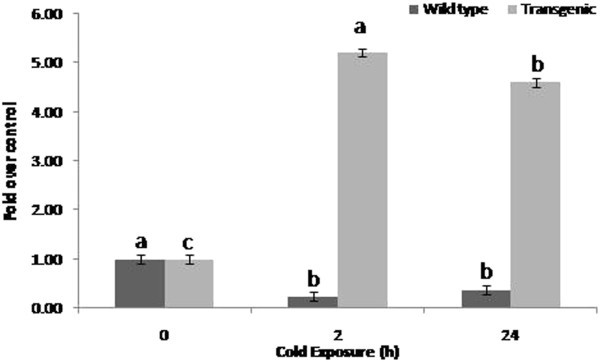
**Effect of cold exposure on Proline accumulation in transgenic and wild type tomato plants exposed to cold treatment.** Plants at 90 days after transplanting were exposed to cold treatment for 2 and 24 h and proline accumulation was studied in leaves. The values are mean (n = 4) fold Proline accumulation over the respective untreated controls. Error bars indicate SE. The mean values in one series indicated by different letters are statistically significant (P ≤ 0.05) according to LSD test.

Free Proline accumulation reduced significantly in wild type plants on cold (4°C) exposure for 2 and 24 h (Figure 2). However, in transgenic tomato plants, the Proline accumulation was significantly higher (565.4 μg/g FW) on cold exposure of 2 h than that of untreated (108.6 μg/g FW) and cold exposure of 24 h (499.8 μg/g FW). Proline content on 24 h cold exposure was significantly higher than that of untreated control.

**Figure 3:**
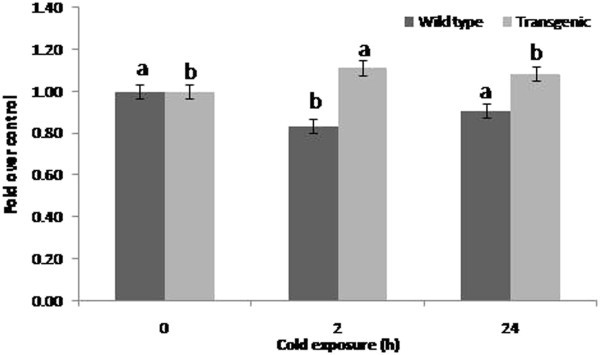
**Effect of cold exposure on ascorbate content in transgenic and wild type tomato plants exposed to cold treatment.** Plants at 90 days after transplanting were exposed to cold treatment for 2 and 24 h and ascorbate content was analyzed in leaves. The values are mean (n = 4) fold ascorbate content over the respective untreated control. Error bars indicate SE. The mean values in one series indicated by different letters are statistically significant (P ≤ 0.05) according to LSD test.

Ascorbate content on 2 h cold exposure was significantly lower (3.87 μg/g FW) than untreated control (16.86 μg/g FW) in wild type plants (Figure 3). However, in case of 24 h cold exposure, the ascorbate content was similar to that of untreated control. In case of transgenic, the ascorbate content was significantly higher on cold exposure of 2 h whereas, it was similar to that untreated control on 24 h of cold exposure.

## Discussion

Plants are often subjected to various biotic and abiotic stresses because of changes in the environmental conditions, leading to a series of morphological, physiological, biochemical and molecular changes, adversely affecting its survival, growth, and productivity. Results of our earlier containment and laboratory studies of *Osmotin* transgenic tomato have established cold tolerance in the transgenic. Further, physio-biochemical analyses suggested better antioxidant defence (through higher antioxidant enzyme activity) and osmotic adjustment (through accumulation of Proline) in the transgenic than the wild type on cold exposure (Goyary 2009). Plants alter expression of regulatory cold responsive genes or the genes encoding functional proteins (the enzymes involved in the synthesis of osmoprotectants and antioxidant defence) in response to cold exposure. However, regulation of these genes in *Osmotin* transgenic plants is not yet studied. Therefore, in the present study, transcript expression of the genes involved in various functions including transcript regulation (*CBF1*) osmotic adjustment (*P5CS*), and antioxidant defence (*APX*) along with *Osmotin* was analyzed using qRT PCR to understand the molecular mechanisms of cold tolerance in the transgenic tomato. Transcription factors as well as most of the other early expressing genes undergo rapid induction in transcript expression in response to short (15 min - 6 h) exposure to specific stimuli and expression is again reduced on long term exposure, similar to that of basal level (Patade et al. 2012). Whereas, number of effector genes normally take relatively longer exposure (6–24 h) to stimuli for their induction in transcript expression. Therefore, in the present study, in order to study the regulation of stress responsive early expressing as well as the effector genes, the transcript expression was studied in response to short (2 h) and relatively long term (24 h) cold (4°C) exposure.

Stimulated induction of transcript expression of *Osmotin* gene is reported in response to biotic as well as abiotic stimuli (Zhu et al. 1995a, b). Moreover, its increased transcript abundance through constitutive over expression of the gene in transgenic plants has resulted in improvement in cold and other abiotic stress tolerance (Goyary 2009; Parkhi et al. 2009; Goel et al. 2010; Das et al. 2011; Subramanyan et al. 2011). In the present study, transcript accumulation of *Osmotin* gene was significantly higher in transgenic tomato in response to 2 h of cold exposure. Thus, the cold tolerance in the *Osmotin* transgenic tomato was associated with early up regulation of *Osmotin* transcript expression. On the other hand, the expression level of *Osmotin* gene in wild type tomato decreased on cold exposure. Thus, cold sensitive response in wild types may be attributed to the reduced transcript abundance of *Osmotin* transcripts.

Of the numerous transcription factors involved in cold signalling, ICE-CBF-COR is the most studied and important transcriptional cascades, as it plays vital role in protecting plants from deleterious effects of cold stress (Thomashow 2010). C-Repeat binding factors (CBFs), a family of transcription factors, regulates the expression of COR genes on cold stress exposure. Constitutive or stress-inducible over expression of *CBF1* in transgenic plants has enhanced chilling and freezing stress tolerance in various crops including tomato (Hsieh et al. 2002) and rice (Lee et al. 2004) indicating a pivotal role of *CBF1* in gene regulation during cold acclimation in evolutionarily diverse plant species. In the present study, both transgenic as well as wild type tomato displayed accumulation of *CBF1* transcripts in response to cold treatment. However, the extent of increase in transcript abundance was significantly higher in transgenic (36 fold) than that in wild type (19 fold).

The results of numerous earlier studies have indicated osmotic as well as other potential roles of Proline in protecting cellular structure or ROS detoxification. Induction of transcript expression of gene involved in Proline biosynthesis (Δ1-pyrroline-5-carboxylate synthase- *P5CS*) has been reported in response to abiotic stresses (Neffar et al. 2011; Zhuang et al. 2011). Further, over expression of the gene has resulted in enhanced Proline accumulation and presumably, the stress tolerance (Vendruscolo et al. 2007; Sharma et al. 2011). However, the functions depend on spatial and temporal regulation of its transcript expression and further metabolism to meet the plant’s needs (Sharma et al. 2011). In the present study, transcript expression of *P5CS* gene as well as Proline content was induced in transgenic plants on 2 h cold exposure. However, the transcript expression as well as the Proline content was significantly reduced in wild types on cold exposure. Our earlier biochemical analyses have also revealed significantly increased free Proline content in transgenic tomato than the wild types on cold exposure (Goyary 2009). In addition, the similar results have also been reported by independent biochemical analyses in *Osmotin* transgenic tomato and other plants on salt and drought stress exposure (Parkhi et al. 2009; Goel et al. 2010; Barthakur et al. 2001). Nevertheless, whether the systematic fall in the abundance of *P5CS* transcript in wild type plants over the entire duration of cold stress has any metabolic or adaptive significance needs to be investigated in depth.

ROS accumulated in response to various stresses are counteracted by intrinsic antioxidant systems including enzymatic scavengers like ascorbate peroxidase (APX). In plants, APX plays crucial role in the removal of H_2_O_2_. In the present study, the transcript expression of the cytosolic *APX* gene as well as the ascorbate content induced earlier in response to 2 h cold exposure in transgenic tomato. Whereas, in wild type tomato plants, the induction in transcript expression was delayed and induced only on 24 h cold exposure. The ascorbate content analysis in wild type plant also revealed reduced ascorbate content on 2 h of cold exposure whereas on 24 h of cold exposure, the ascorbate content was similar to that of untreated control. Baek and Skinner ( 2003) have reported induced transcript expression of thylakoid bound *APX* during cold acclimation in near-isogenic lines (NILs) of wheat, differing in the *Vrn1-Fr1* chromosome region that conditions winter versus spring wheat growth habit. Similarly, Lin and Pu ( 2010) reported increased transcript accumulation of cytosolic *APX* in salt tolerant sweet potato cultivar than the sensitive. The earlier induction in transcript expression of *APX* as well as the ascorbate content in *Osmotin* transgenic tomato than the wild type could have resulted in better antioxidant defense, as revealed in our earlier experiments, leading to cold tolerance (Goyary 2009).

## Conclusions

The transcript expression of the selected stress responsive genes was differentially regulated in the *Osmotin* transgenic and wild type tomato plants on short and relatively long term cold exposure (Table 1). The transcript expression on cold exposure either induced earlier or the extent of increase was higher in the transgenic tomato plants than that of wild type. The metabolite analyses on free Proline accumulation and ascorbate content also supported the transcript expression data. As the gene products are involved in various stress alleviation functions such as transcript regulation of target stress responsive genes, osmotic adjustment, and antioxidant defence; the altered transcript regulation and the metabolite contents could be responsible for the tolerant response in the transgenic plants. Thus, the results suggests that constitutive over expression of *Osmotin* in tomato modulate expression of other stress responsive genes thereby imparting cold tolerance.

**Table 1:** **Summary of transcript regulation of genes over the respective untreated controls**

Gene	Transcript regulation on cold exposure over the respective untreated controls			
	2 h		24 h	
	Wild type	Transgenic	Wild type	Transgenic
*Osmotin*	Down^b^	Up^a^	Down^c^	Down^c^
*CBF1*	Up^a^	Up^a^	Unchanged^b^	Up^b^
*P5CS*	Down^b^	Up^a^	Down^c^	Unchanged^b^
*APX*	Unchanged^b^	Up^a^	Up^a^	Up^b^

The transgenic and wild type tomato plants were exposed to cold treatment for 2 and 24 h. The significant (P ≤ 0.05) differences in transcript regulation (up, down or unchanged) among the cold and control treatments are marked with different letters.

## Methods

### Cold treatment

Seeds of *Osmotin* transgenic (T7) tomato (cv. Pusa Ruby) were sown in germination trays in containment to get plants of advanced (T8) generation. At the same time, seeds of tomato cv. Pusa Ruby were sown to get wild type plants. Seedlings were transplanted in pots filled with potting mixture (sand: soil: FYM; 1:1:1) 40 days after sowing (DAS).

The grown up transgenic and wild type plants (4 per treatment) in reproductive growth stage (at 90 days after transplanting) were exposed to cold treatment (4°C) for 2 and 24 h in a cooling incubator equipped with cold fluorescent light with adjusted photoperiod of 16 h.

### RNA isolation and first strand cDNA synthesis

All the glassware and plastic-ware used in RNA isolation were treated with 0.1% (v/v) Diethyl pyrocarbonate (DEPC; SIGMA chemicals, USA) for 24 h followed by autoclaving for 1 h and oven drying at 80°C for 48 h to make RNase free. Total RNA was isolated from leaf samples harvested from all the treatments using TRIzol reagent (SIGMA chemicals, USA) as per earlier optimized protocol (Patade et al. 2012). The quality and quantity of the isolated total RNA was checked by absorbance at 260 and 280 nm.

First strand cDNA was synthesized from the isolated RNA (2.0 μg) using oligo (dT) primer (500 ng). To the RT-PCR reaction, dNTPs (final concentration 2 mM), M-MuLV reverse transcriptase (200 U), 5X assay buffer, RNasin (40 U) and DTT (final concentration 5 mM) were added and the reaction volume was made up to 20 μl with nuclease free water. The reaction was carried out at 42°C for 1 h in a thermal cycler (Bio-Rad S1000, Singapore). The cDNA was tested for amplification using *18S rRNA* specific primers for 35 cycles.

### qRT PCR

Genes specific Primers (Table 2) were designed using Primer3 software (Rozen and Skaletsky 2000) based on the sequences available at NCBI GenBank database (http://www.ncbi.nlm.nih.gov). The reaction mixture was prepared using 2 × SYBR Green Quantifast mix (Qiagen, USA), gene specific primers (10 pM), cDNA as a template and nuclease free molecular biology grade water. The SYBR Green master mix contained pre-optimised ROX as passive reference dye. The thermal cycling programme consisted initial denaturation (95°C, 7 min), followed by 30 cycles of denaturation (95°C, 30 sec), primer annealing (60°C, 30 sec) and primer extension (72°C, 30 sec). The qRT PCR reactions were carried out in a real time thermal cycler-Max3005P (Stratagene, Germany). For ascertaining equal RNA loading in RT reaction, *18S rRNA* was used as an internal control and the fold expression of the target genes was normalized. The transcript expression of the selected stress related genes was quantified based on C_T_ values using ΔΔ _CT_ method (Livak and Schmittgen 2001).

**Table 2:** **Primers designed for transcript expression analysis of stress responsive genes**

Sr. No.	Primer Id	GenBank Accession No.	Forward Primer (5’-3’)	Reverse Primer (5’-3’)
1.	*Osmotin*	X61679.1	AATGCGCCACGAGGTACTAA	AGGACTCCACCACAGTCACC
2.	*CBF1*	AY368483.1	GGCTAATGTTACCACCACCTCAA	CAATAATTCGCACCATACCCAAA
3.	*P5CS*	NM_001246978.1	TGCACTGGAAGCAAATGAAA	CCATCAGCAATCTCCGTTCT
4.	*APX*	X81376.1	CCTCTTATGCTCCGTCTTGC	CTCCAGTCACCTCAACAGCA
5.	*18S rRNA*	BG130735.1	AGGGACTACGGCCTTTTAGG	CAGAAGGGGACAATTTCAAAGA

The qRT PCR products were further directly separated on agarose gel (2%) and the mobility of the amplicons was captured based on the fluorescence emitted using a Phosphorimager (Typhoon 9410, GE Healthcare, USA) with emission filter: 610 BP30 (Deep Purple, SYPRO, Ruby, EtBR) at 488 nm, with focal plane adjusted +3 mm above the surface (Additional file 1: Figure S1). The captured 1D gel images were further analyzed using ImageQuant^TM^ TL software (GE Healthcare, USA).

### Free Proline accumulation and ascorbate content

Free Proline content was determined on four biological replicates, according to Bates et al. ( 1973). Leaf samples (200 mg) were homogenized in aqueous sulfosalicylic acid (3% w/v; 3 ml). The filtered homogenate (2 ml) was reacted with equal volume each of acid ninhydrin and acetic acid at 100°C for 1 h and the reaction was terminated in an ice bath. The reaction mixture was extracted with 4 ml toluene and mixed vigorously with a stirrer for 10-15 s. The chromophore containing toluene was aspirated from the aqueous phase and warmed to room temperature. The absorbance was recorded at 520 nm using toluene as a blank. Proline concentration (μg g^-1^ FW) was determined from a standard curve prepared with 10 standard concentrations (0-50 μg) of L-Proline. The fold Proline content over the respective untreated controls in wild type and transgenic was then calculated.

Ascorbate contents were measured using procedure described by Kampfenkel et al. ( 1995) with slight modification. Ground leaf tissue (250 mg) was extracted with 600 μl of ice-cold 6% trichloroacetic acid (TCA), and was incubated on ice for 15 min followed by centrifugation for five min at 15,000 × *g* at 4°C. The supernatant (200 μl) was then mixed with 600 μl of 0.2 M phosphate buffer (pH 7.4), and 200 μl of double distilled water. The mixture was then treated with 1000 μl of 10% (w/v) TCA, 800 μl of 42% (v/v) o-phosphoric acid, 800 μl of 4% (w/v) a,a’-dipyridyl (dissolved in 70% [v/v] ethanol), and 400 μl of 3% (w/v) FeC1_3_. Mixtures were incubated in a water bath at 42°C for 40 min and subsequently the absorbance was read at 525 nm. Blank reaction with 6% TCA instead of samples was used for correcting the color development in absence of sample. Known concentrations of ascorbate (Himedia) dissolved in 6% (w/v) TCA were used for standard curve preparation to measure the ascorbate content (μg g^-1^ fresh weight) in the samples. The fold ascorbate content in wild type and transgenic over the respective untreated controls was then calculated.

### Statistical analysis

RNA was isolated from four plants of each treatment and further two technical duplicates were considered in Real Time PCR for individual cDNA sample. Similarly, free Proline accumulation and ascorbate content was analyzed from leaves of four plants of each treatment. CropStat program (IRRI, Philippines) was used for analysis of variance (ANOVA) of experiments laid out in a completely randomized design.

## Electronic supplementary material

Additional file 1: Figure S1: Gel images showing transcript abundance of in wild type (W) and *Osmotin* transgenic (T) tomato plants in response to cold treatment. Plants at 90 days after transplanting were exposed to cold treatment for 2 and 24 h and transcript expression was studied in leaves. Fluorescence of SYBR Green 1 dye bind with real time PCR products was documented in Phosphorimager (Typhoon Scanner, Model Typhoon TRIO+, GE HealthCare, USA) with 610 BP30 emission filter and blue laser. PCR products obtained for the internal control (18S rRNA) were also visualized for data normalization. (PPTX 238 kb) (PPTX 239 KB)
