# Supplementary material for: Cold tolerance in Osmotin transgenic tomato (Solanum lycopersicum L.) is associated with modulation in transcript abundance of stress responsive genes
Source: Springerplus. 2013 Mar 19;2:117. doi: 10.1186/2193-1801-2-117 (PMC3610025; doi:10.1186/2193-1801-2-117)
Supplement: Supplementary file 1 — Additional file 1: Figure S1: Gel images showing transcript abundance of in wild type (W) and Osmotin transgenic (T) tomato plants in response to cold treatment. Plants at 90 days after transplanting were exposed to cold treatment for 2 and 24 h and transcript expression was studied in leaves. Fluorescence of SYBR Green 1 dye bind with real time PCR products was documented in Phosphorimager (Typhoon Scanner, Model Typhoon TRIO+, GE HealthCare, USA) with 610 BP30 emission filter and blue laser. PCR products obtained for the internal control (18S rRNA) were also visualized for data normalization. (PPTX 238 kb) (PPTX 239 KB) [file 40064_2012_173_MOESM1_ESM.pptx]

## Slide 1
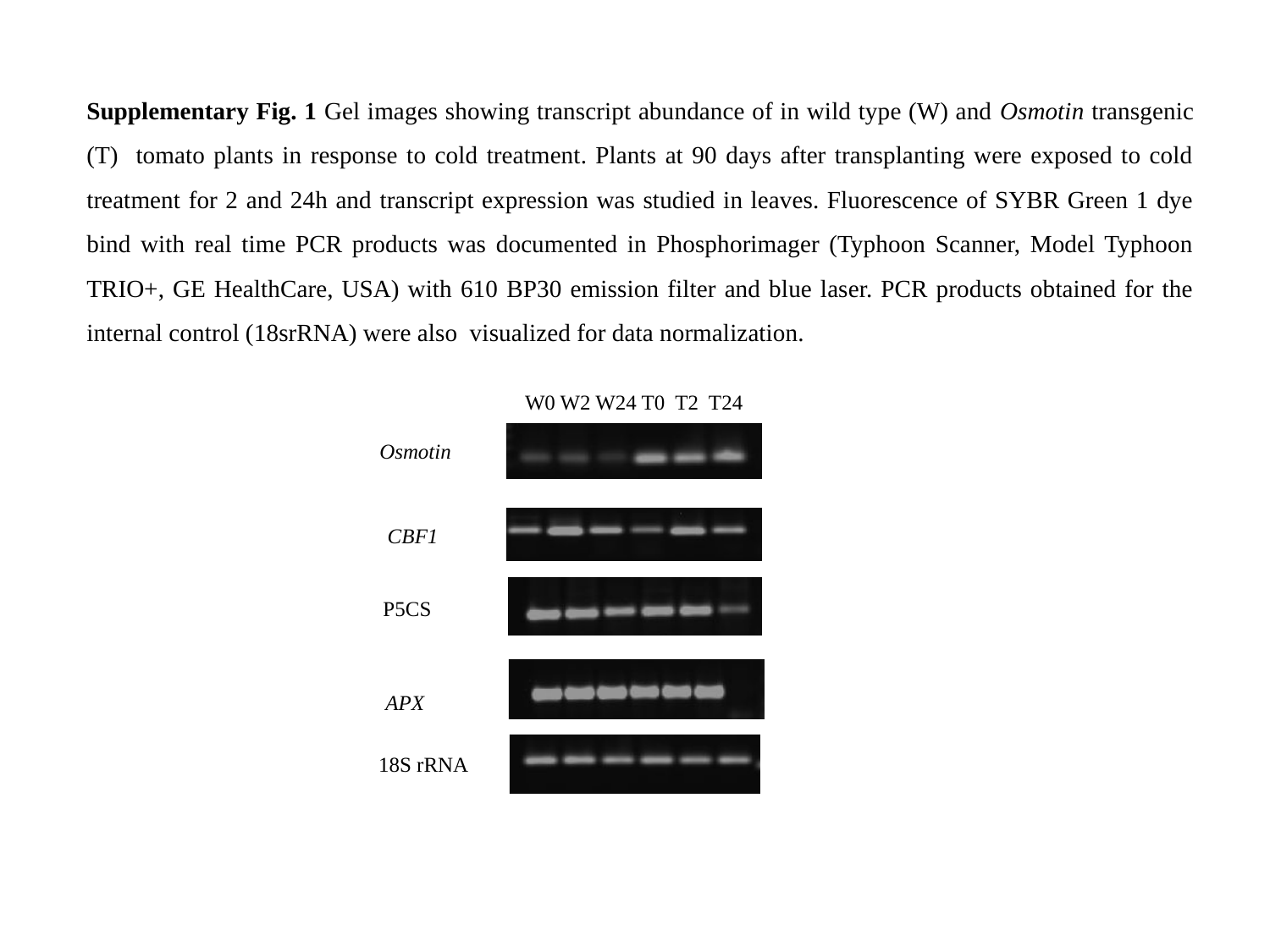

Supplementary Fig. 1 Gel images showing transcript abundance of in wild type (W) and Osmotin transgenic (T) tomato plants in response to cold treatment. Plants at 90 days after transplanting were exposed to cold treatment for 2 and 24h and transcript expression was studied in leaves. Fluorescence of SYBR Green 1 dye bind with real time PCR products was documented in Phosphorimager (Typhoon Scanner, Model Typhoon TRIO+, GE HealthCare, USA) with 610 BP30 emission filter and blue laser. PCR products obtained for the internal control (18srRNA) were also visualized for data normalization.
 W0 W2 W24 T0 T2 T24
# Osmotin
CBF1
P5CS
APX
18S rRNA
